# Uniform intensity in multifocal microscopy using a spatial light modulator

**DOI:** 10.1371/journal.pone.0230217

**Published:** 2020-03-11

**Authors:** M. Junaid Amin, Sabine Petry, Haw Yang, Joshua W. Shaevitz

**Affiliations:** 1 Department of Molecular Biology, Princeton University, Princeton, New Jersey, United States of America; 2 Department of Chemistry, Princeton University, Princeton, New Jersey, United States of America; 3 Lewis-Sigler Institute for Integrative Genomics, Princeton University, Princeton, New Jersey, United States of America; 4 Department of Physics, Princeton University, Princeton, New Jersey, United States of America; Nicolaus Copernicus University, POLAND

## Abstract

Multifocal microscopy (MFM) offers high-speed three-dimensional imaging through the simultaneous image capture from multiple focal planes. Conventional MFM systems use a fabricated grating in the emission path for a single emission wavelength band and one set of focal plane separations. While a Spatial Light Modulator (SLM) can add more flexibility as a replacement to the fabricated grating, the relatively small number of pixels in the SLM chip, cross-talk between the pixels, and aberrations in the imaging system can produce non-uniform intensity in the different axially separated image planes. We present an in situ iterative SLM calibration algorithm that overcomes these optical- and hardware-related limitations to deliver near-uniform intensity across all focal planes. Using immobilized gold nanoparticles under darkfield illumination, we demonstrate superior intensity evenness compared to current methods. We also demonstrate applicability across emission wavelengths, axial plane separations, imaging modalities, SLM settings, and different SLM manufacturers. Therefore, our microscope design and algorithms provide an alternative to the use of fabricated gratings in MFM, as they are relatively simple and could find broad applications in the wider research community.

## Introduction

Multifocal microscopy is a useful method that allows simultaneous imaging of multiple object planes to realize high-speed 3D imaging. In general, this technique allows imaging of multiple axially separated object planes simultaneously in a single camera exposure of a 2D CMOS or CCD imaging sensor. This is commonly done by directing the sample scattered or fluorescence emission through a custom designed grating, known as a multifocal grating. The multifocal grating is uniquely patterned such that it generates multiple diffraction orders, with each order having a unique degree of defocus associated with it. Each diffraction order is then imaged side by side onto a camera sensor to result in a 3D image where different regions of the sensor, which are denoted as subimages in this paper, correspond to different object planes. There is no motion of sample or objective to obtain 3D images. In such systems, up to a hundred or more 3D sample volumes per second can be recorded since the only hardware limiting factor to the 3D volume imaging speed is the frame rate of the camera. The imaging process is also simultaneous which means all the 2D-sectioning planes are imaged/monitored at the same time leading to minimal loss of 3D sample information. These characteristics make multifocal imaging an attractive avenue for researchers.

There are several implementations of the multifocal microscope in literature: Blanchard et al. [1] used a custom fabricated ‘quadrically distorted’ multifocal grating patterned using an algorithm based on the detour phase effect [2]. S. Abrahamsson et al. [3] used a custom fabricated grating patterned using a similar detour phase effect based algorithm [2] as used in [1], though with the presence of more precise defocus terms and aberration correction optics for large emission bandwidth as well as high NA (up to 1.4) fluorescence imaging. They present a software-based ‘Pixelflipper’ method to optimize the fabricated multifocal grating patterns intended to achieve illumination evenness across the subimages in their multifocal microscope. The method in [3] has been adapted in other microscope modalities such as polarization microscopy [4] and structured illumination microscopy [5]. Fabricated gratings, however, are limited to a fixed Δz value, where Δz is the object space separation between the different object planes being imaged simultaneously. Creating such gratings necessitates access to fabrication facilities which require extensive training and investment, rendering them out of reach of many research labs. In addition, changing the Δz value in real time is not possible without replacing differently designed custom gratings. A new grating needs to be designed and fabricated for every wavelength and every unique object spacing Δz, which is another time and resource consuming process.

The fabricated grating can be replaced with a Liquid Crystal Spatial Light Modulator (SLM). SLMs are pixelated liquid crystal devices with programmable control on the relative phase of light striking each pixel. Deploying SLMs as multifocal gratings has numerous advantages: they are available off the shelf, require no additional investment other than the initial cost and, most importantly, they can dynamically change the phase mask to adapt to multiple wavelengths or object plane separations requirements [6,7]. However, uniform illumination across the subimages is difficult to achieve using an SLM-based phase mask due to inherent device characteristics including pixel-to-pixel crosstalk effects [8,9]. These SLM hardware-related issues prevent the use of prior-art uniform illumination methods such as the Pixelflipper and Iterative Fourier Transform algorithms to be successfully applicable to SLMs. We present an in situ iterative calibration method for the generation of optimized SLM phase patterns that produce multifocal images with near-uniform subimage brightness.

## Background

### Prior-art methods for obtaining uniform subimage brightness in multifocal microscopes

**Fig 1:**
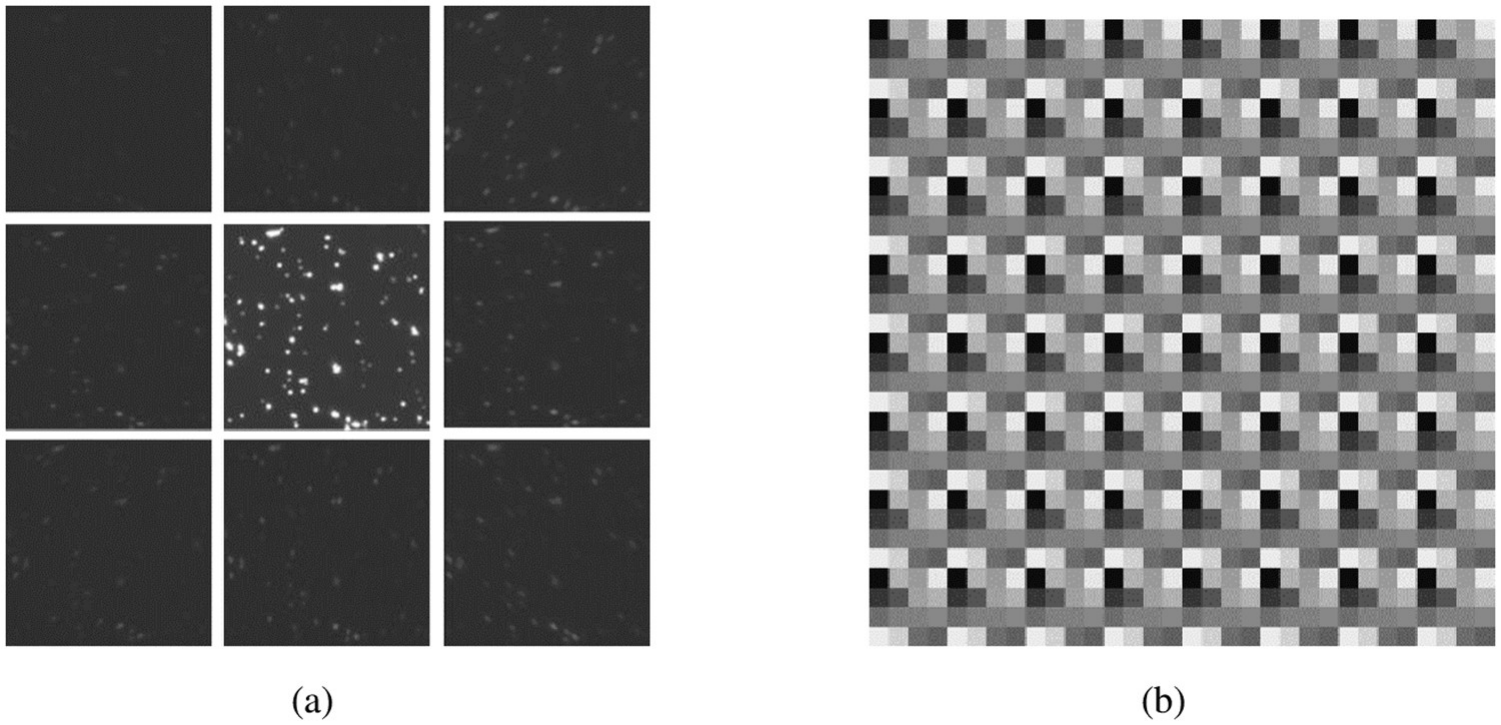
(a) In-focus subimages cropped from a 9 plane z stack acquired from the camera resulting from deploying a Pixelflipper algorithm optimized SLM display pattern. The emission light is unevenly distributed in the subimages, with the central zeroth order subimage receiving most of the emission light, and (b) zoomed-in view of a 64 × 64 pixels^2^ region of the SLM displayed grating pattern which gives the multifocal subimages in (a).

The phase grating in a multifocal microscope has two tasks: (i) to divide incoming emission light equally into a 2D array of diffraction orders, and (ii) to axially offset the orders to different object planes separated by a distance *Δz* through modifying the phase pattern using a geometric distortion function. The Pixelflipper algorithm was specifically designed to generate uniformly-illuminated subimages in existing multifocal microscope systems [3]. This algorithm uses a software-based method that finds the phase pattern of a grating unit cell of size *P*_*u*_ × *P*_*u*_ pixels^2^ that gives the highest uniformity among the diffraction orders in the computed Fourier plane. This optimized unit cell is then repetitively arranged into a grid to provide the phase pattern to be displayed on the SLM. A more general algorithm used to obtain SLM phase patterns given a desired far-field intensity distribution is the Iterative Fourier Transform Algorithm (IFTA) [10]. Both Pixelflipper and IFTA often fail to produce adequate results using SLMs as it assumes an aberration free optical system that takes the Fourier transform precisely. Furthermore, in addition to other system aberrations, SLMs suffer from pixel-to-pixel crosstalk effects [8,9] that further alter the resultant diffraction pattern. This issue is worsened particularly when only few SLM pixels form the repeated grating pattern. These effects are illustrated in the image of a 100 nm Gold Nanoparticles (AuNPs) under darkfield illumination on an SLM-based multifocal microscope (see S1 File) using *Δz* = 0 nm (Fig 1(a)). There is an undesirably significant intensity difference among subimages even though the Pixelflipper algorithm was used to optimize the SLM pattern with *P*_*u*_ = 4 and G = 80 where G denotes the discrete number of gray levels in the phase pattern spread over the 8-bit addressing range of the SLM (Fig 1(b)).

## Methods

### In situ iterative calibration routine for subimage intensity uniformity

Here, we propose an in situ iterative calibration method to generate phase patterns which allows near-uniform illumination in the subimages of an SLM-based multifocal microscope. The algorithm is based on a feedback loop between the SLM, camera, and the computer, and uses real-time images from the camera to update the pattern on the SLM until the optimal grating pattern is acquired. To evaluate the subimage intensity uniformity for our optimization routine, we modified the metric proposed in ref. [11] and used instead the following metric which also includes a background subtraction term,

$$M=\frac{\text{min}\left(\left\{I_{m,i}\right\}\right)-I_{b}}{\text{max}\left(\left\{I_{m,i}\right\}\right)-I_{b}},$$ (1)

where {*I*_*m*,*i*_}is the measured subimage *i* (*i* = 1 … *N*×*N*) and *I*_*b*_ is a measured background intensity. *M* ranges from 0 to 1, with *M* = 1 corresponding to completely uniform subimage intensities.

**Fig 2:**
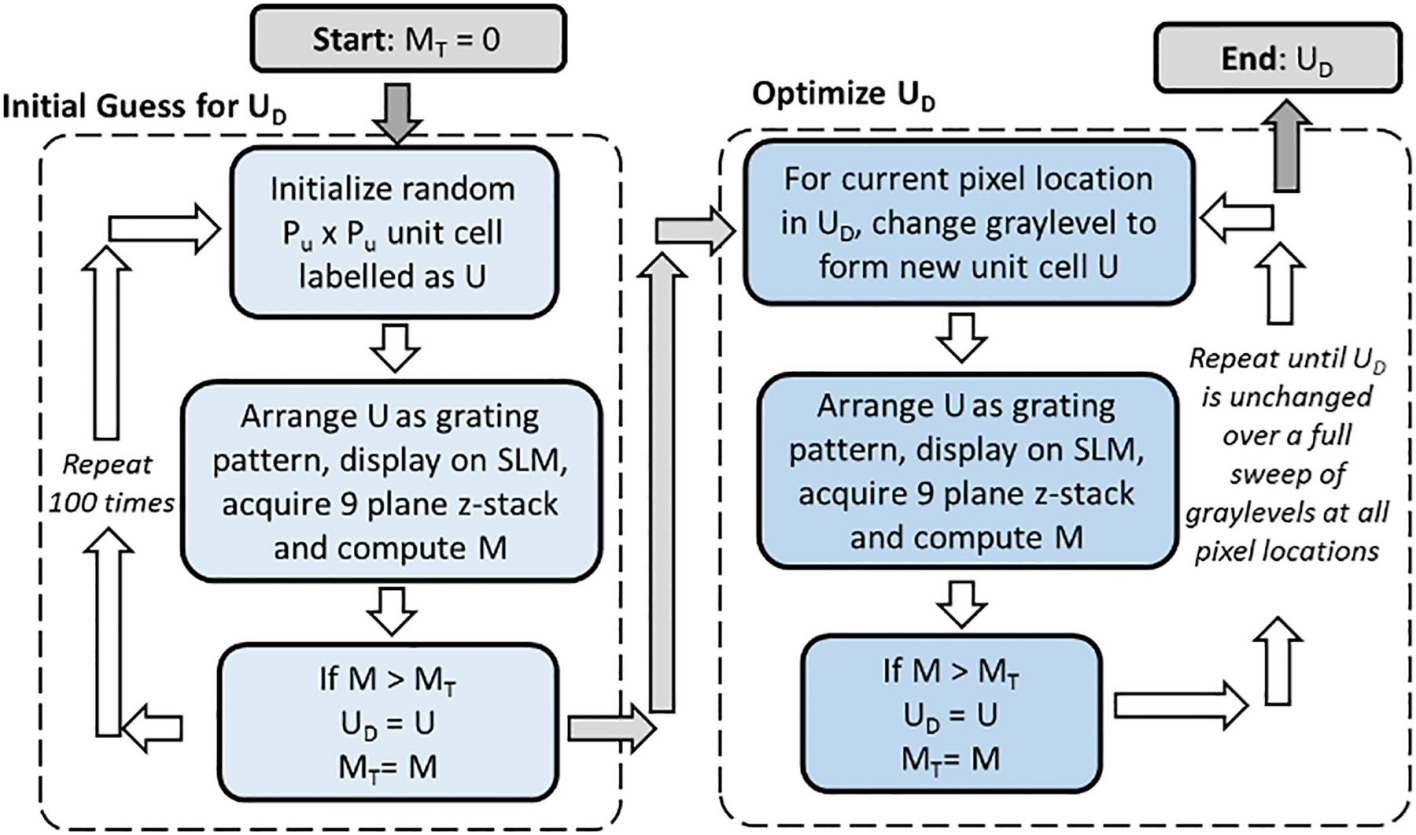
Block diagram illustrating in situ iterative algorithm implementation.

The in-situ iterative algorithm block diagram is shown in Fig 2. Note that all *M* values in this method are evaluated from the experimental images obtained in real-time. To begin, an initial *M* value threshold *M*_*T*_ is set to zero and input into the “Initial Guess” stage. In this stage, the unit cell, denoted as *U*_*D*_, having the highest *M* is chosen from 100 different randomly generated unit cells, with *M*_*T*_ becoming this corresponding *M* value. *U*_*D*_ and *M*_*T*_ are then sent into the “Optimization” stage where *U*_*D*_ is optimized iteratively to maximize *M* by sequentially iterating over all available graylevel values and for all pixel locations spanning the unit cell. This grid search routine is repeated until there is no change in *U*_*D*_ throughout a complete iteration over all pixel locations and graylevel values. This concludes the algorithm, with *U*_*D*_ being the output of the in situ iterative procedure.

## Results

**Fig 3:**
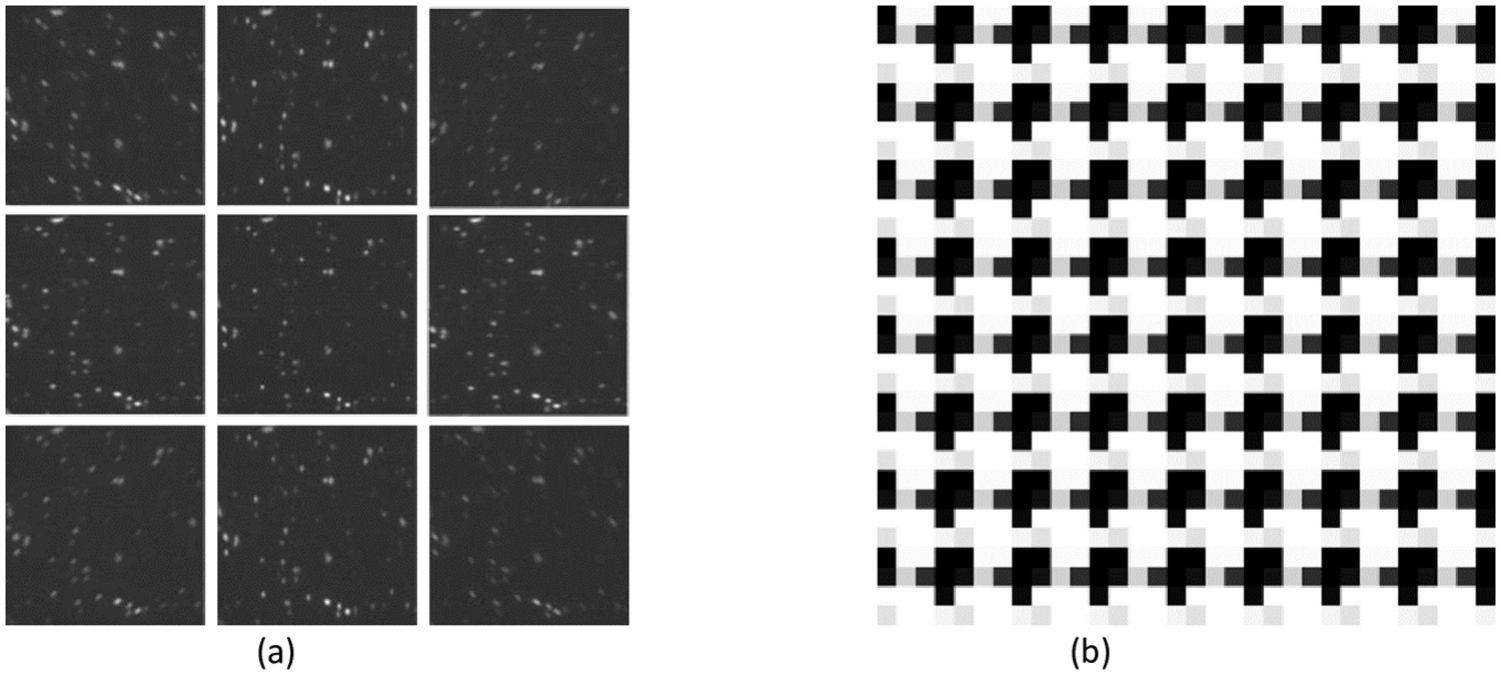
(a) In-focus subimages cropped from a 9 plane z stack acquired resulting from deploying our in situ iteratively optimized SLM display pattern. The emission light striking the camera is more evenly distributed among the subimages compared to Fig 1(a), and (b) zoomed-in view of a 64 × 64 pixels^2^ region of the SLM displayed grating pattern which gives the images in (a).

**Fig 4:**
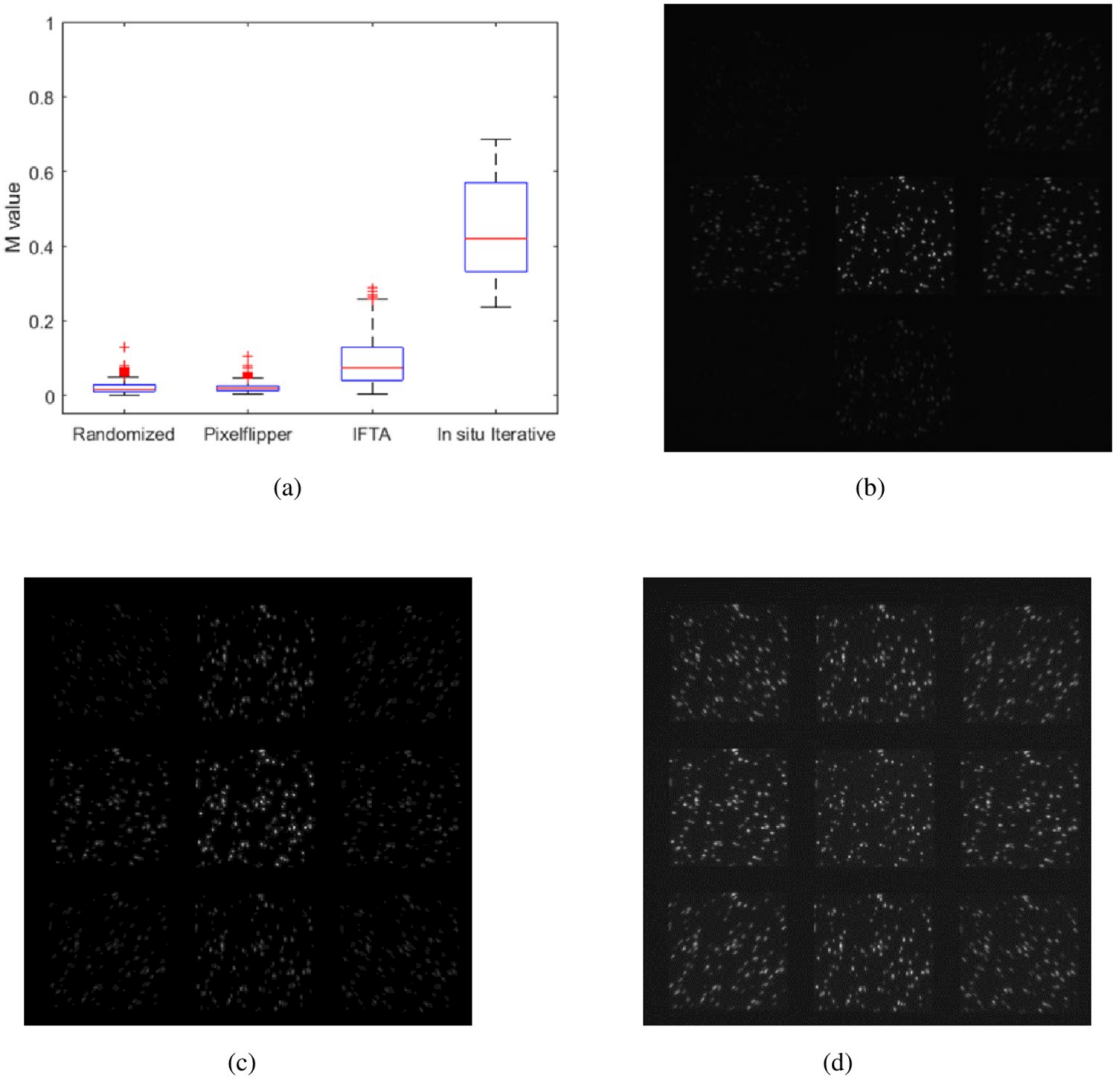
(a) Boxplots of output M values resulting from grating patterns optimized using Pixflipper (500 iterations, Randomized (500 iterations), IFTA (500 iterations) and our in situ iterative algorithm (12 iterations), (b) image resulting from Pixelflipper algorithm generated pattern having *M* = 0.062, (c) image resulting from IFTA algorithm generated pattern (*M* = 0.211), and (d) image resulting from in-situ iterative algorithm generated pattern (*M* = 0.644).

The in situ iterative method gives visually uniform subimage intensities using *Δz* = 0 nm (Fig 3(a)), when displaying an in situ iterative optimized pattern on the SLM (Fig 3(b)). The computed *M* value for this pattern is 0.712, much larger than the *M* value measured when the Pixelflipper-based phase mask is used (*M* = 0.033, Fig 1(a)). We compared multiple trials of the Pixelflipper algorithm, our in situ iterative method, IFTA and randomly-generated phase patterns using P_u_ = 4 and G = 80(Fig 4(a)). Sample images resulting from Pixelflipper, IFTA and in-situ iterative methods are displayed in Fig 4(b), 4(c) and 4(d), respectively. The in situ iterative method shows superior performance over both the Pixelflipper and the randomized methods, realizing multifocal images with large *M* values, i.e., near-uniform subimage intensities (Fig 4).

### Multifocal imaging of biological specimen

**Fig 5:**
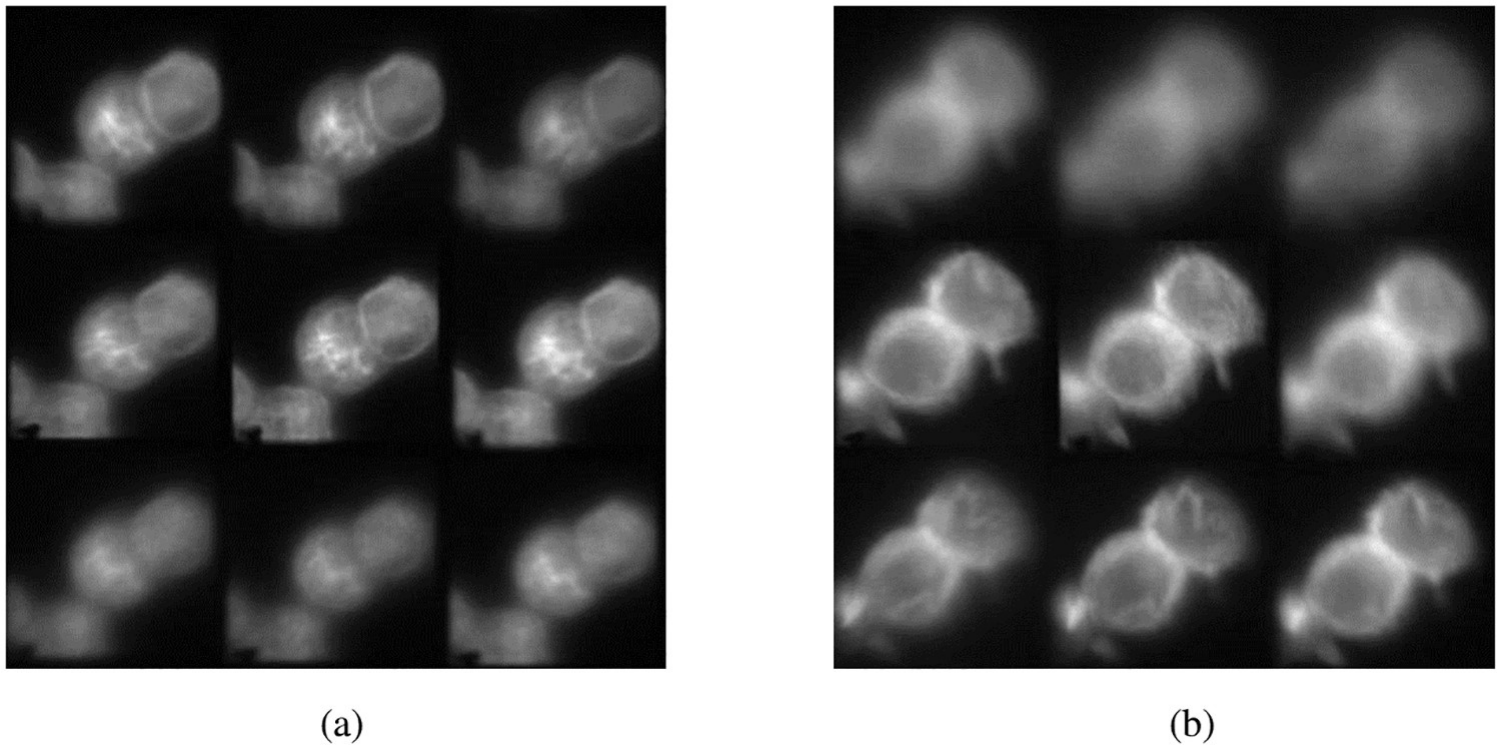
Multifocal images of different regions of a microtubule stained Hela Kyoto Cells fixed sample with (a) Δz = 0.50 μm, and (b) Δz = 1.00 μm.

We used our in situ iteratively optimized SLM phase patterns to acquire 3D images of GFP-labeled tubulin in MeOH-fixed TPX2 Hela Kyoto cells [12]. The in situ iterative calibration algorithm was first executed using darkfield imaging under the current settings to obtain the optimized SLM phase pattern, which was then phase distorted using the algorithm in [3] to achieve object plane separation in the subimages. Multifocal snapshots of the sample cells using 488-nm laser excitation were obtained (Fig 5). The sequence of the subimages in the multifocal image (Fig 5) follows the sequence shown in S1(b) Fig in S1 File, with the top right plane of the image corresponding to the *z* = -4*Δz* plane, where *Δz* represents the focal plane separation in object space. The *Δz* values for the images (Fig 5) are 0.50 μm (Fig 5(a)) and 1.00 μm (Fig 5(b)). The different focal cross-sections of the cells can be visibly seen across the subimages, displaying the structural features in the sample and the 3D imaging power of the multifocal microscope.

## Discussion

We demonstrate that our in situ iterative optimization algorithm is effective at generating SLM-based patterns which allow uniformity across multifocal subimages. The key underlying concept of the algorithm is the inclusion of real-time experimental variables into the optimization process. Many of the parameters involved such as *P*_*u*_, the emission wavelength and the SLM model are empirical hardware choices. In particular, the *P*_*u*_ = 4 value throughout the paper is empirically chosen to maximize the imaging field of view without overlapping of the subimages in the current system optical settings. Other microscope setups may require a different unit cell size to be optimal. Note that the metric M deployed in this implementation of the in situ iterative algorithm is intentionally designed to optimize the uniformity of the subimages, which has been a challenge thus far in SLM-based MFMs. This metric does not necessarily result in the highest possible diffraction efficiency (see S1 File). Also note that Figs 1(a) and 3(a) are obtained using Δz = 0 nm settings to keep the subimages identical for clear illustration of their subimage illumination uniformity characteristics. In-situ-iterative optimized patterns are empirically found to exhibit identical subimage illumination independent of Δz settings.

Deploying SLMs as an alternative to multifocal gratings in MFMs has numerous advantages: they are available off the shelf, require no additional investment other than its initial cost, and can readily be programmed to change any multifocal grating parameter including Δz values as well as number of simultaneous imaging planes at high speed, limited by the refresh rate of the SLM (typically 60 Hz). On the other hand, the relatively large SLM pixels may limit the achievable field of views for each subimage due to a limited range of grating periods, particularly when shorter visible wavelengths are deployed. Additionally, SLMs suffer from optical losses, including loss of half the emission intensity at the polarizer located in front of the SLM. This loss could be avoided by deploying a technique developed by Backlund et. al [13]. Fabricated gratings, on the other hand, have numerous advantages. They allow much smaller effective pixel sizes arising from the fabrication process, resulting in more control over the shape and period of the phase mask. However, each mask is limited to a fixed Δz value and wavelength range. Furthermore, such manufactured gratings require access to clean room facilities having fabrication and lithography tools which may not be readily available near many research labs. Nevertheless, fabricated grating based MFMs continue to be an important tool for researchers. Using our in situ iterative algorithm, researchers now have an alternative option to build SLM based MFMs for investigating fast microscopic 3D processes in biology, physical chemistry and other domains. The broad applicability of the calibration routine is demonstrated to account for different SLM manufacturers, wavelength, unit cell sizes as well as different microscope modalities (see S1 File), making this method applicable to varying imaging requirements. Future work involves exploring various other optimization techniques and metrics to further improve subimage intensity uniformity, diffraction efficiency and optical throughput in the SLM based MFM.

## Supporting information

S1 File (DOCX)
