# Supplementary material for: Uniform intensity in multifocal microscopy using a spatial light modulator
Source: PLoS One. 2020 Mar 11;15(3):e0230217. doi: 10.1371/journal.pone.0230217 (PMC7065765; doi:10.1371/journal.pone.0230217)
Supplement: S1 File — (DOCX) [file pone.0230217.s001.docx]

Supplementary

1. SLM based Multifocal Microscope (SLM-MFM)

1.1. Optical Diagram

Fig. S1(a) illustrates the optical design of the home-built multifocal microscope. Three main segments of the system are highlighted for easier visualization: Darkfield-Brightfield (DF-BF) illumination, laser illumination and multifocal Imaging. In the DF-BF illumination


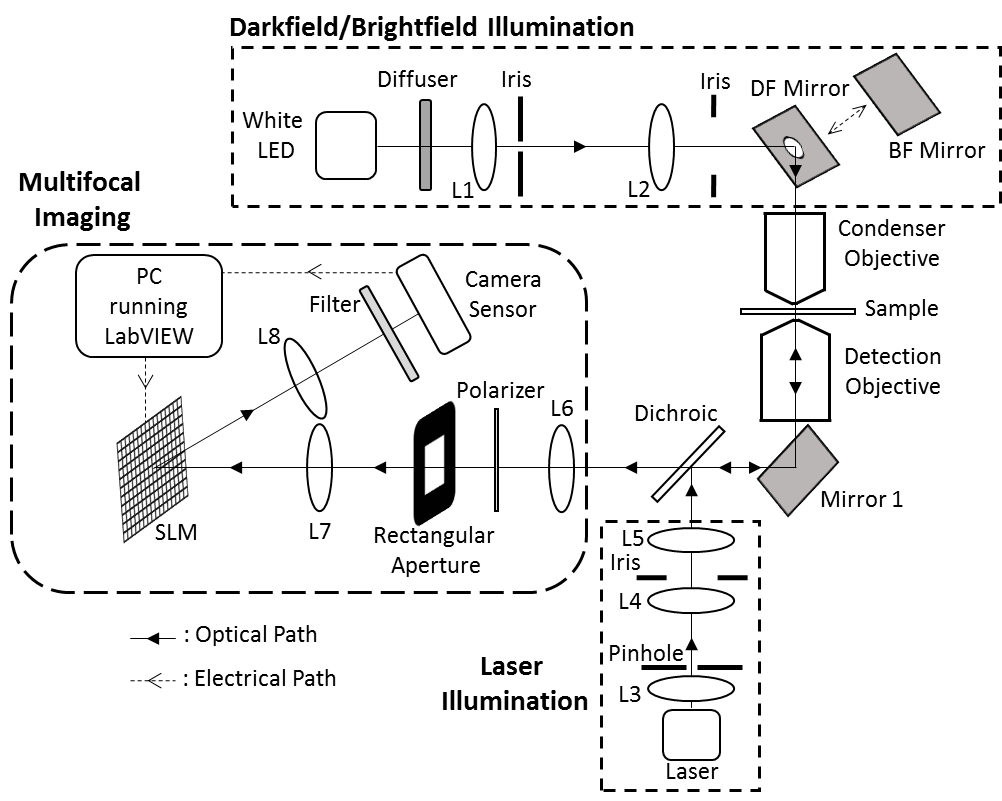

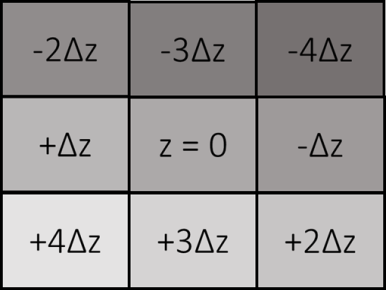


(a) (b)

Fig. S1. (a) Optical diagram of the SLM based Multifocal Microscope, and (b) front view of the camera sensor showing the placement of the simultaneously sub-images, each corresponding to a unique object plane.

module, incoherent light from a white LED passes through a Diffuser before being focused by a lens L1 onto an iris. Lens L2 then collimates the light and sends it onto the DF Mirror to realize Darkfield Imaging mode. The DF Mirror is a custom designed mirror with an oval central transmissive area thereby allowing reflection of the incoming light into a ring-shaped intensity pattern downwards into the condenser objective. To switch from Darkfield mode to Brightfield imaging mode, the DF Mirror can be replaced by the BF Mirror, where the BF Mirror is a conventional fully reflecting optical mirror. Note that when operating in Darkfield mode, the Detection Objective’s Numerical Aperture (NA) is kept smaller than that of the Condenser Objective.

In the laser illumination module, a laser is spatially filtered and collimated by a combination of lens L3, a Pinhole and lens L4. Lens L5 is introduced to focus the beam reflecting off a dichroic to the back focal plane (BFP) of the detection objective for epi-illumination. This beam will be used for excitation of fluorescent samples. Note that although fluorescence samples can also be used for the experimental calibration routine, they aren’t optimal due to bleaching induce changing intensities in the images relative to more stable darkfield and brightfield imaging modes.

In the multifocal imaging module, lens L6 functions as a tube lens and forms an image of the sample at the plane of the rectangular aperture through a linear polarizer. The function of the rectangular aperture is to control the Field of View (FOV), thereby preventing the obtained subimages from overlapping at the imaging plane. The polarizer is necessary for the phase-only function of the deployed SLM. Lens L7, having a focal length f_7_, is placed a focal length’s distance from the rectangular aperture, while the reflective SLM is located at the Fourier plane of L7. With the focal lengths of L6 and L7 the same, the SLM is essentially conjugate to the BFP of the detection objective which is also the Fourier plane of the sample.

The SLM acts as a multifocal grating in this setup. Each of the diffraction orders emanating from the SLM displayed multifocus grating pattern is encoded with a unique defocus phase (except the unaffected zeroth order). These orders when imaged onto the camera sensor, by Lens L8 (having focal length f_8_) via an emission filter, form a multifocus image. This image contains N x N subimages with each subimage represents a unique object plane. Details of the algorithm used to generate such grating patterns are described later in the paper. The bandwidth of the emission filter is restricted to around ~15 nm to reduce chromatic dispersion effects originating from the SLM displayed grating. This emission wavelength band restriction can be overcome using chromatic correction optics as used in [3], but is not implemented here. Fig. S1(b) illustration shows the front view of the camera in the multifocus microscope designed for a 3 x 3 array of subimages, though in theory any N x N number of orders can be obtained. Different segments of the camera in Fig. S1(b) correspond to different object planes, with each subimage separated by a distance ∆z in object space. Note that the zeroth order remains undiffracted and unaffected by the SLM displayed pattern, and thus corresponds to the z = 0 plane. A Personal Computer (PC) running LabVIEW Software interfaces with the SLM and the camera sensor.

For the SLM-MFM, it useful to know the effective lateral FOV as a function of different microscope parameters involved. FOV in this paper is defined as the field of view for each subimage in sample space. To find the expression for FOV, denote Mag as the combined magnification of the detection objective and lens system, P_u_ the SLM displayed grating period in pixels units, S the SLM pixel size and λ_min_ the minimum wavelength in the emission band. The angle θ between the zeroth and 1^st^ orders of a grating is found using the grating equation: θ = sin^-1^[λ_min_/(P_u_ x S)]. Once θ is known, the FOV can be equated by finding the distance between the centers of both zeroth and 1^st^ orders on the image plane, before dividing by the Mag:

$FOV=\left(\frac{1}{Mag}\right)f_{8}\tan\left(\theta\right)$, (S1)

1.2. Experimental Setup

The SLM-MFM microscope is custom built in the lab to test the effectiveness of grating patterns designed to optimally distribute incoming light equally into the diffraction orders. For the Darkfield imaging mode, Thorlabs Solis-3C High-Power LED is deployed as the white light LED, along with the accompanying DC20 driver module for intensity control. The diffuser used is Thorlabs DG20-1500. Lens L1 is Thorlabs LA1401-A (focal length = 60 mm) and L2 is AC508-150-A-ML (focal length 150 mm). The DF mirror is custom designed to match the dimensions of the condenser objective used which is the MPLAN BD 50x NA 0.75 objective from Olympus. The detection objective is a Leica 100x, NA 1.4 - 0.7, where the NA is set to 0.7 during Darkfield imaging. For the sample, a mixture is formed using 10 uL of stock 100 nm Gold nanoparticles (AuNPs) solution from BBI solutions and 10 uL of 1M NACl solution. 10uL is ejected onto a 22 × 22 mm^2^ coverslip (Fisher scientific) using a pipette before being covered directly by an 18 × 18 mm^2^ coverslip. The salt is added to immobilize the AuNP onto the coverslip surface. Nail Polish from Electron Microscopy Sciences is used to seal the coverslip edges to avoid leakage of the solution. The sample is mounted onto a P-611.3S NanoCube XYZ Piezo stage from Physik Instrumente using custom machined mounts. Both the piezo stage and the detection objective are mounted on a custom designed aluminum block, which forms the microscopy body. The condenser objective is screwed to a Newport 460A-XYZ translation stage via a custom aluminum adapter plate, with this stage mounted on an 8 inch high post to position the condenser above the sample. Lens L6 is Thorlabs AC508-200-A-ML (focal length = 200 mm), while both f_7_ and f_8_ are set to 200 mm (Thorlabs AC254-200-A-ML). The polarizer model is LPVISE100-A, the rectangular aperture is model # 61-1137 from Ealing Catalog, USA, while the emission filter deployed is Semrock FF01-685/10-25 with a central wavelength of 685 nm and FWHM ~ 15 nm. For the biological experiment, the emission filter is changed to one with a 510 nm center wavelength having a bandwidth of ~16 nm. The camera sensor is Hamamatsu’s Orca-Flash4.0 V3 sCMOS with a pixel resolution of 2048 x 2048 pixels and a pixel size of 6.5 μm. The camera exposure time is set to 250 ms for the duration of the experiment, and all images are stored as raw 16-bit “.tif” format.

The SLM deployed is the reflective Holoeye PLUTO-2-VIS-056 Phase-only spatial light modulator. It has an 8-bit pixel display resolution, a 1920 x 1080 array of pixels and a pixel pitch S = 8 μm. It can be calibrated, i.e., mapping of displayed gray-level images to actual phase imparted to the incoming light, using different methods. One method is to use the manufacturer provided calibration data specified for certain wavelengths which can be loaded directly onto the SLM via its USB port. Another method involves an experimental procedure outlined in the manufacturer’s manual which involves striking the two halves of the SLM with circular top-hat beams originating from the same laser and observing the resulting interference pattern. This latter method is not easily applicable in multifocal setups since the SLM is used in the emission path with emission wavelengths over the visible range. Acquiring lasers for each desired emission wavelength is expensive and impractical, with no guarantee of being effective for a spread of wavelengths as is the case here. Therefore, for this experiment, a default manufacturer-provided calibration curve meant for a 2.2π phase cycle corresponding to 0 – 255 graylevel values for 633 nm laser is uploaded to the SLM firmware. This gives an approximately 2π phase cycle for 696 nm, closely matching the current emission wavelength centered at 685 nm. Apart from the calibration, an important step is to ensure that all SLM displayed patterns have an aperture similar to that of the BFP of the detection objective. Thus, all displayed patterns are multiplied by an aperture function with the central region size corresponding to the size of the BFP. Furthermore, the region outside this aperture is set to a tilted grating with an empirically chosen defocus pattern to steer any stray incoming emission light striking outside the SLM main aperture area away from the zeroth order. Therefore, any subsequent grating patterns will only be displayed inside the central region of the aperture function.

With the experimental setup arranged as described, we implement the prior-art Pixelflipper algorithm described in Section 3.1 using LabVIEW software. N is chosen to be equal to 3, giving a target matrix T of size 3 × 3. With 256 SLM displayable graylevels at our disposal, we limited the graylevel resolution to 80 steps spanning the 0–255 range to reduce the algorithm run time which empirically provides similar performance to having 256 graylevel steps. The Pixelflipper algorithm is run by setting P_u_ = 4 to give an optimized matrix unit cell U of dimensions 4 × 4. This U is then arranged in a grating format and is phase ‘distorted’ to give a ∆z value of 0.90 µm to realize multifocus imaging, before being multiplied by the aperture function described earlier. Once this resulting pattern is displayed on the SLM, a 9 plane z stack (i.e., images of the sample obtained at multiple z positions by vertical motion of the piezo stage) of the sample is obtained, where the axial spacing of the z stack is chosen to match the deployed ∆z = 0.90 µm.

To find the value of M due to a given grating pattern using Eqn. (1), the following procedure is followed: a particle of interest in the field of view is selected and an 80 × 80 pixels^2^ area around that particle is chosen as the Region of Interest (RoI). For each subimage *i*, where *i* is an integer between 1 and 9, the mean intensity $I_{m,i}$of its respective RoI is calculated from the image when it is in focus. For example, the mean intensity of subimage 1, $I_{m,1}$, is found by processing only the plane of the z stack when subimage 1 is in focus. Whereas, the mean intensity of, e.g., subimage 7, $I_{m,7}$*,* is found by processing only that plane of the z stack when subimage 7 is in focus. Once the mean intensities $\left\{ I_{m,i} \right\}$of all subimages are found, the minimum and maximum $\left\{ I_{m,i} \right\}$values are selected for use in Eqn. (1). To calculate $I_{b}$, a uniform graylevel pattern is displayed on the SLM which results in the SLM not directing light into the subimages, except into subimage 5 which is the zeroth order and receives most of the incoming light. In this setting, the mean intensities calculated over the same ROIs for all subimages other than subimage 5 are calculated and denoted as $\left\{ I_{b,j} \right\}$ where *j* is an integer between 1 and 9, other than 5. $I_{b}$ is then defined as the minimum value among $I_{b,j}$. In this step, note that the minimum of $I_{b,j}$ is chosen as $I_{b}$, and not the average of $I_{b,j}$, to avoid negative M values which can occur when min($\left\{ I_{m,i} \right\}$) – $I_{b}$ is negative where $I_{b}$ is brighter than min($\left\{ I_{m,i} \right\}$). Therefore, with min($\left\{ I_{m,i} \right\}$), max($\left\{ I_{m,i} \right\}$) and $I_{b}$ at hand, Eqn. (1) is used to compute M. Furthermore, the algorithm takes around 15 minutes to execute when using P_u_ = 4 and G = 80 gray levels spread over the 8-bit addressing range of the SLM phase pattern. This time frame will change when different values of the parameters P_u_ and G are used.

2. SLM-MFM subimage field flatness

The SLM-MFM simultaneously images multiple object planes into, e.g., 3 × 3 array of subimages. In addition to optimizing the intensity distribution among the subimages, it is also beneficial to characterize the field flatness of the subimages as a result of the phase distortion implemented in the SLM grating pattern to achieve the 3D imaging capability. An understanding of the uniformity across each subimage is a necessary step in interpreting and processing multifocal 3D imaging data acquired from this microscope.

The following procedure is deployed to characterize the field flatness across the subimages in Darkfield imaging mode: A solution of 200 nm fluorescent beads (660/680) from Life Technologies Corporation is diluted an empirically chosen 400 times before being mixed with a solution of 1 Molar NaCl in a 1:1 volume ratio. 10 µL of this mixture is ejected on a coverslip (22 × 22 mm^2^ coverslip from Fisher scientific) using a pipette before being covered with another coverslip (18 × 18 mm^2^ coverslip (Fisher scientific)). The edges of the smaller coverslip are sealed with nail polish (Electron Microscopy Sciences). As before, the salt helps to immobilize the fluorescent beads to the coverslip surface. The sample is mounted on the piezo stage and a 647 nm Cobolt 130 mW CW laser is used for illumination in an epi-configuration. On the emission side, a ~3nm emission window is created by inserting both Semrock filters FF01-685/10-25 and FF01-685/LP-25 into the light path. The ~3 nm bandwidth significantly minimizes the chromatic dispersion in the subimages (other than the zeroth order subimage 5 which is unaffected). This prevents dispersion related aberrations from negatively affecting the bead localization process described shortly. For illustration,




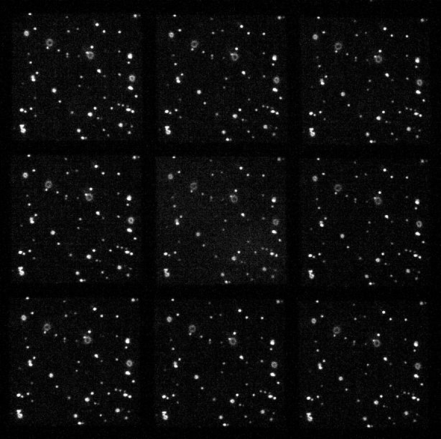


(a) (b)

Fig. S2. (a) Images of 100 nm immobilized AuNPs acquired under Darkfield Illumination using emission filter bandwidths of (a) ~ 15 nm, and (b) ~ 3 nm.

multifocal images acquired using ~3 nm and ~15 nm emission filter bandwidths is shown in Fig. S2. Z-stacks are acquired using an in situ iteratively optimized pattern displayed on the SLM having ∆z values of 0 µm and 1.00 µm, using stage stapes of 20 nm. For each z-stack, the locations of the beads within the field of view are identified in the lateral and axial Cartesian coordinates. The lateral (*xy*) positions are identified by identifying the bright regions in a focal projection of the z-stacks using the *imfindcircles()* function in MATLAB. Appropriate radii and intensity thresholds are applied to remove possible bead aggregates. The axial (*z*) location of the beads are identified by computing the maximum of the Brenner gradient in a square 16 × 16 pixels^2^ region around each identified bead for all images in the z-stack. This localizes the beads in the axial direction.

Once the lateral and axial positions of the beads are found for each subimage, they are plotted in 3D and the *xz* views are displayed in Fig. S3. Fig. S3(a) plot shows displays field flatness data acquired using ∆z = 0 µm, whereas Fig. S3(b) corresponds to data acquired when ∆z = 1.00 µm. These plots demonstrate near uniform fields across all subimages. Even without correcting for possible sample tilt inherent to the setup, the average peak-valley (P-V) value among subimages acquired using ∆z = 0 µm is 0.665 µm while the mean P-V value for ∆z = 1.00 µm is 0.467 µm, both well within 2% variation across the field of view signifying reasonably flat subimages most practical multifocal imaging purposes.


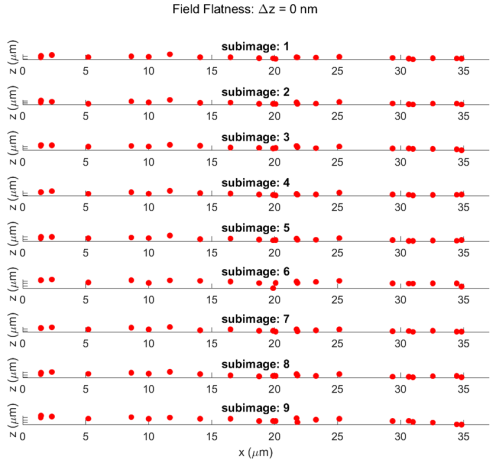

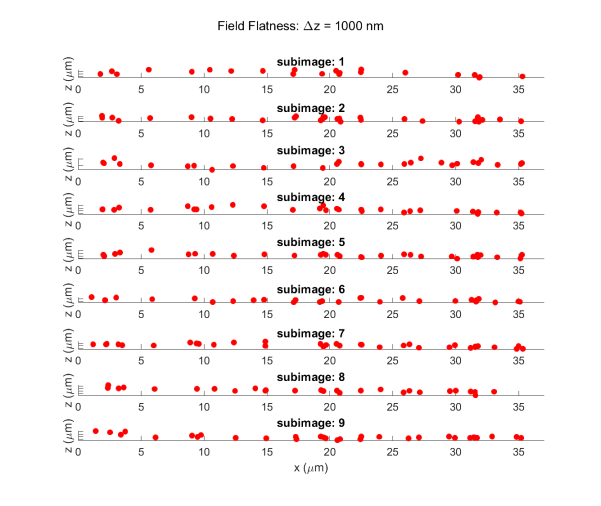


(a) (b)

Fig. S3. xz view of the xyz localization of 200 nm beads immobilized on a coverslip to demonstrate the field flatness of the 9 subimages for ∆z values of (a) 0 µm, and (b) 1.00 µm.

3. Uniform illumination of orders using Brightfield Imaging mode

To engage the Brightfield imaging mode, the DF Mirror in Fig. S1(a) is replaced by the BF Mirror to allow full reflection of the light incoming from L2. In this arrangement, we first remove the sample completely, and allow the unscattered light focused by the condenser passes through the detection objective and towards the multifocal optics. Another possibility is to have a cut-out piece of S4 paper as the sample to act as a scattering object. Both methods are tested to work adequately. Apart from the Imaging mode, other key parameter changes for this demonstration includes setting ∆z = 0 µm. Since ∆z = 0, there is no need to take z-stacks for M value calculation, therefore a single image is obtained for each pattern and the sub-images are processed from it. In addition, M values calculated using the Brightfield imaging mode are denoted M_BF_, to discriminate from M calculated in Darkfield imaging mode. Another parameter change involves setting f_8_ to 100 mm, which has no effect on the order illumination distribution characteristics but changes the magnification Mag by half.

This arrangement is first tested for a pattern generated by the Pixelflipper using the unchanged P_u_ = 4 and G = 80. An image acquired using this Pixelflipper optimized pattern is shown in Fig. S4(a). A purple box is shown to annotate the camera region covered spanning the 3 × 3 orders. Fig. S4(b) shows 64 × 64 pixels^2^ region in the SLM displayed Pixelflipper optimized patter. Visually, the illumination spread among the orders, similar to Fig. 1(a) in terms of contrast, is far from uniform. The computed M_BF_ value for the Fig. S4(a) image is 0.126.


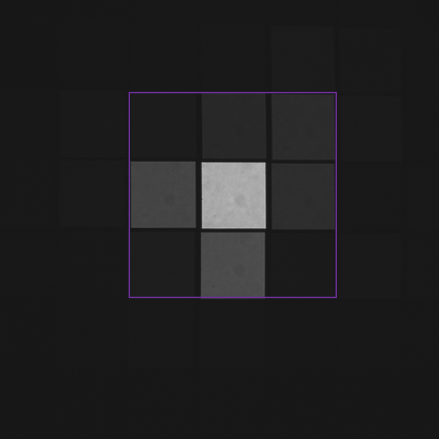

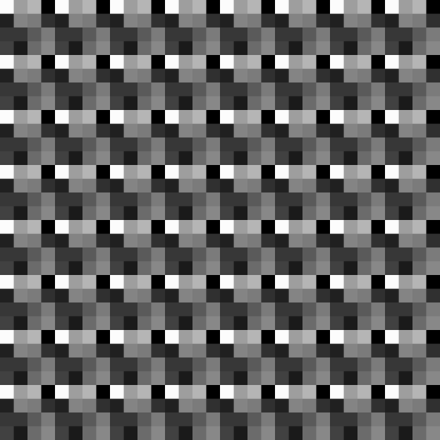


(a) (b)


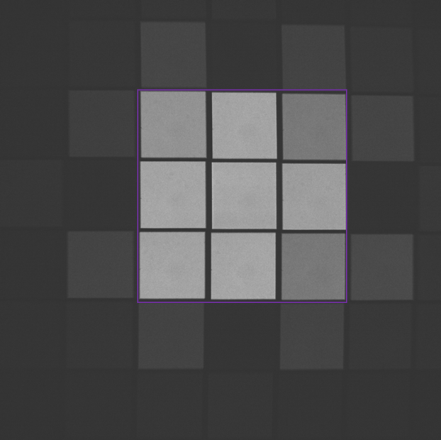

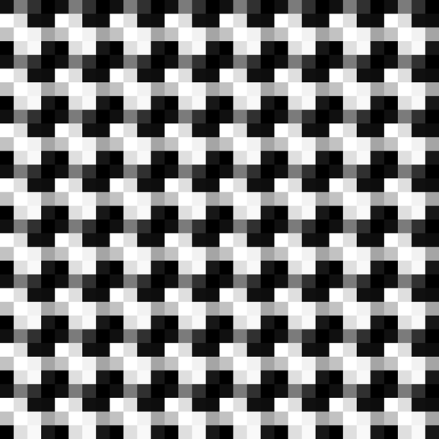


(c) (d)

Fig. S4. Using the Brightfield Imaging mode, (a) image resulting from deploying a Pixflipper optimized SLM displayed pattern. The pattern is intended to give a 3 x3 array of uniformly illuminated subimages. The M_BF_ value for (a) is 0.126, and (b) zoomed-in view of the SLM displayed grating pattern which gives the image in (a), showing the repetitive arrangement of unit cells,(c) Image resulting from deploying an output optimized pattern from the proposed in situ iterative algorithm. The pattern is intended to give a 3 x3 array of uniformly illuminated subimages. The M value for (a) is 0.712, and (b) zoomed-in view of SLM displayed grating pattern which gives the image in (a), showing the repetitive arrangement of unit cells.

Fig. S4(c) shows an output image due to an in situ iteratively optimized pattern, with the purple box annotating the relevant 3 × 3 sub-images region. A 64 × 64 region of the pattern is shown in Fig. S4(d). Visually, Fig. S4(c) shows a higher uniformity of illumination across the orders. The M_BF_ value for Fig. S4(c) is computed to be 0.712. In terms of statistics, the Pixelflipper and randomized pattern generation algorithm are repeatedly executed 1000 times each, and M_BF_ values are computed for each pattern. These M_BF_ values are displayed as a boxplot in Fig. S5 which also shows M_BF_ values from 30 iterations of our algorithm. This plot demonstrates the high degree of illumination uniformity improvement due to our algorithm.

Note that, in Fig. S4(c), additional subimages apart from the bright 3 × 3 subimage array are also visible. These are additional orders which receive illumination from the grating pattern. In the current algorithm framework, only the intensities of the 3 × 3 subimages are optimized, with no correction for the intensity spilling out into the other orders. In future work, new optimization will be explored which allow high efficiency illumination of the subimages while suppressing the unused diffraction orders’ intensity to a minimum.


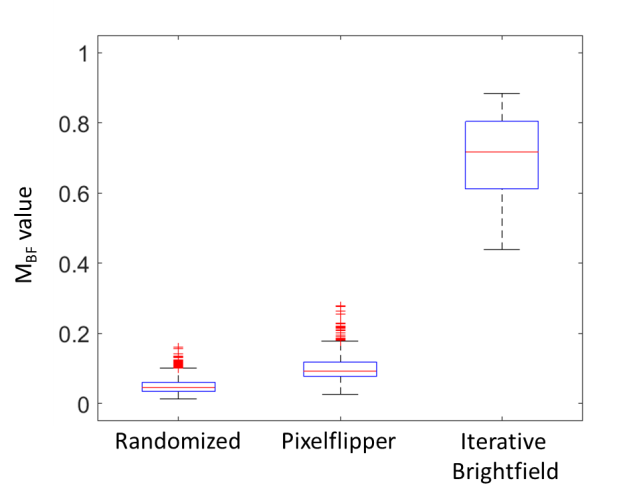


Fig. S5. (a) Boxplots of output MBF values resulting from grating patterns optimized using Pixflipper (1000 iterations, Randomized (1000 iterations) and our in situ iterative algorithm (30 iterations).

4. Comparison of in situ iterative calibration routine in Darkfield imaging mode versus Brightfield imaging mode

To evaluate the performance of P_u_ = 4 patterns optimized using the in situ iterative calibration method in Brightfield mode, denoted iterative brightfield, compared to the P_u_ = 4 in situ iteratively optimized patterns in Darkfield imaging mode, denoted as iterative darkfield, the 30 brightfield optimized patterns are implemented in Darkfield imaging of the same sample used in obtaining the M value data for Fig. 3 (main text). In this demonstration, all the Brightfield patterns are distorted with ∆z = 0.90 µm. The computed M values resulting from using the Brightfield patterns (30 iterations) in this Darkfield imaging mode are compiled into the Fig. S6 boxplot, which also shows the Fig. 3 (main text) M value data (12 iterations) obtained using our algorithm. This plot indicates that both methods are equally effective in optimizing illumination uniformity across multifocal images.


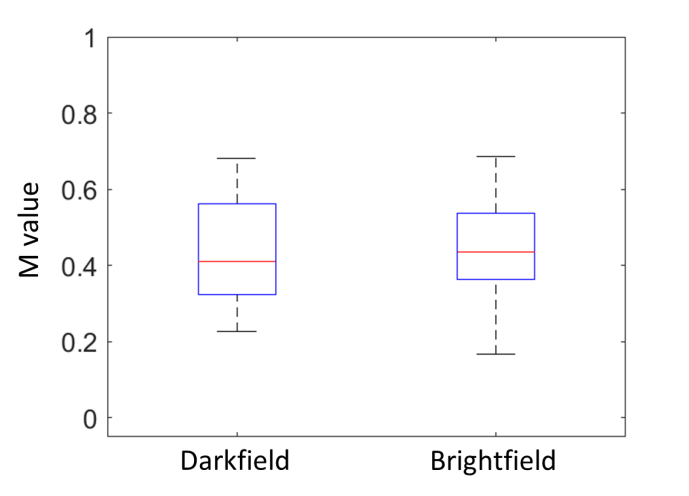


Fig. S6. The boxplot shows a comparison of M values due to 12 different in situ iteratively optimized Darkfield patterns (optimized on AuNP samples under Darkfield imaging) and 30 optimized Brightfield patterns (optimized using Brightfield illumination with no sample) when implemented on the same AuNP sample with ∆z = 0.90 µm.

5. Pixelflipper output using P_u_ > 4

Our calibration method is demonstrated in this paper to significantly improve the intensity distribution in the multi-focus subimages. Prior to this method, the Pixelflipper has been widely applied to fabricated gratings, though with orders of magnitude larger P_u_ values. Due to the large pixel sizes of SLMs, larger P_u_ values equate to large grating periods, which in turn significantly limit the field of view. Selected Pixelflipper algorithm outputs for P_u_ values of 4, 16 and 32 are shown in Fig. S7, which is demonstrated using the Brightfield imaging mode and using f_8_ = 200 mm. Additionally, G is set to 256 for all generated Pixelflipper patterns in this demonstration. Fig. S7(a) shows a camera image captured using P_u_ = 4 using the Pixelflipper optimized pattern whose zoomed in 64 × 64 pixels^2^ region is shown in Fig. S7(b). The FOV for P_u_ = 4 is calculated to be 42.38 µm using f_8_ = 200 mm, Mag = 100, λ_min_ = 678 nm and S = 8 µm. Fig. S7(c) shows a camera image captured using P_u_ = 16, with the zoomed in 64 × 64 pixels^2^ region of the corresponding Pixelflipper optimized pattern shown in Fig. S7(d). In this P_u_ setting of 16 which realizes larger grating periods displayed on the SLM, the diffraction angle is decreased and the FOV decreases by a factor of 4 to 10.60 µm. In between Fig. S7(a) and Fig. S7(c), the Rectangular Aperture is adjusted to prevent overlap between the subimages on the camera sensor resulting from the P_u_ increase. Next, P_u_ is set to 32 and a Pixelflipper optimized pattern is obtained. Fig. S7(e) shows the resulting camera image captured, while Fig. S7(f) shows the zoomed in 64 × 64 pixels^2^ region of the Pixelflipper optimized pattern. As before, the Rectangular Aperture is adjusted to prevent overlap between the subimages on the camera sensor resulting from the P_u_ increase from 16 to 32. The FOV in this P_u_ = 32 setting is now 5.30 µm. Qualitatively, according to Fig. S7, the intensity uniformity among the 9 subimages does improve by increasing P_u_ from 4 to 32. However, this comes at a high cost of eightfold decrease in the FOV, making P_u_ = 32. Furthermore, P_u_ > 32 values are needed to achieve better uniformity, at the cost of further reduction of the FOV. This shows the power of our proposed algorithm which allows high illumination even using P_u_ = 4, without compromising on the FOV.


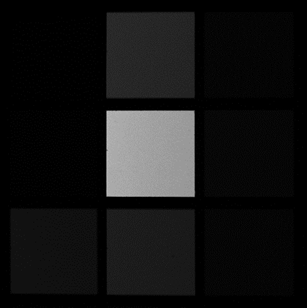

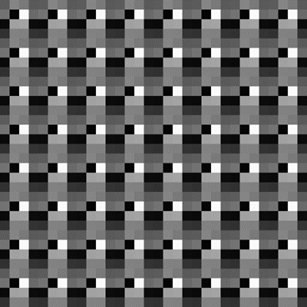

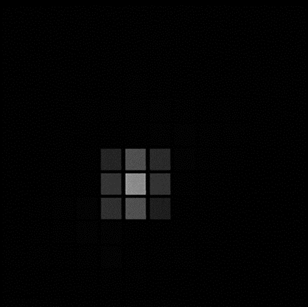


(a) (b) (c)


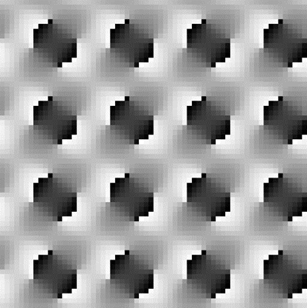

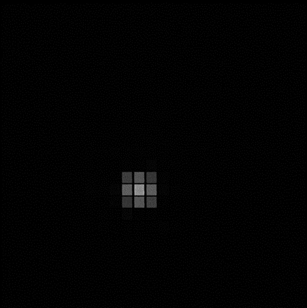

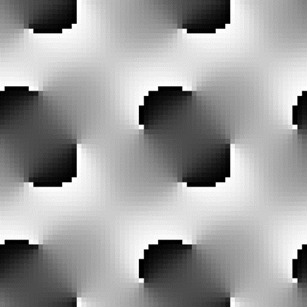


(d) (e) (f)

Fig. S7. In Brightfield Imaging mode, the plots show a comparison of chosen Pixflipper algorithm output patterns for each P_u_ = 4, P_u_ = 16 and P_u_ = 32. (a) The camera image resulting from a P_u_ = 4 Pixflipper optimized pattern, (b) a zoomed in 64x64 pixel^2^ region of the P_u_ = 4 resulting pattern displayed on the SLM, (c) the camera image resulting from a P_u_ = 16 pixflipper optimized pattern, (d) a zoomed in 64x64 pixel^2^ region of the P_u_ = 16 resulting pattern displayed on the SLM, (e) the camera image resulting from a P_u_ = 32 pixflipper optimized pattern, and (f) a zoomed in 64x64 pixel^2^ region of the P_u_ = 32 resulting optimized pattern displayed on the SLM.

6. In situ iterative calibration routine implementation on a different SLM

To demonstrate the universality of our algorithm across SLMs, the Holoeye Pluto-VIS-056 SLM is replaced with a Hamamatsu X10468-07 LCOS-SLM. P_u_ = 4 is used. The X10468 has a larger pixel pitch of 20 µm and has a pixel resolution of 800 × 600 pixels. The Brightfield imaging mode is deployed, and the same algorithm parameters, including P_u_ = 4, is used for this demonstration with the results summarized in Fig. S8. Fig. S8(a) is an example output image due to a Pixelflipper optimized SLM pattern. Note that due to the 20 µm pixel pitch value of this SLM versus the 8 µm of the SLM used earlier, the diffraction angle is smaller bringing the orders closer together on the imaging sensor; therefore the Rectangular Aperture in the optical path is adjusted to prevent the FOVs of the subimages from overlapping with each other. Fig. S8(b) is an output image due to our algorithm optimized pattern, showing a clear increase in the illumination uniformity across the central 3 × 3 orders, in comparison to Fig. S8(a). Fig. S8(c) shows boxplots of M_BF_ values due to the Pixelflipper (90 iterations) and our in situ iterative calibration routine (40 iterations) algorithms, demonstrating the superior performance and applicability of the algorithm. Fig. S8 illustrates the effectiveness of our routine in overcoming the hardware related issues of the Hamamatsu SLM to provide near-uniform intensity spread across the multifocal subimages.


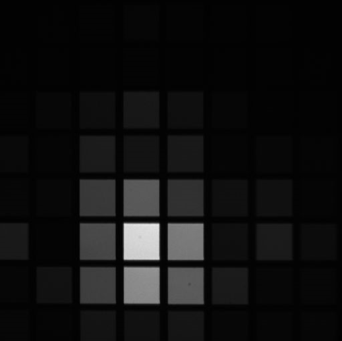

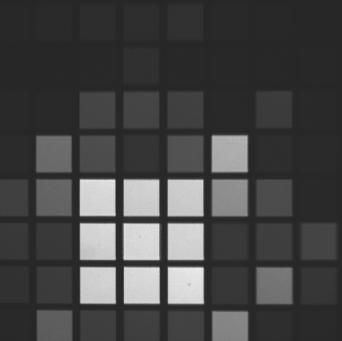

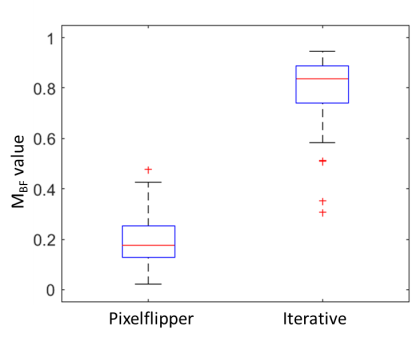


(a) (b) (c)

Fig. S8. Optimization results after replacing the Holoeye Pluto-VIS-056 SLM with the Hamamatsu X10468-07 SLM using P_u_ = 4. (a) output image due to a chosen Pixelflipper algorithm optimized SLM pattern, (b) output image due to our algorithm optimized SLM pattern, and (c) boxplots of M values resulting from 90 iterations of the Pixelflipper and 40 iterations of the our algorithm output. The plot in (c) demonstrates the superior illumination intensity distribution performance of our in situ iterative algorithm.

7. Wavelength dependence of in situ iteratively optimized patterns

The calibration routine patterns optimized for a specific wavelength band are empirically found to give different intensity distributions in the subimages at a different wavelength band. This is demonstrated in Brightfield imaging mode and illustrated in Fig. S9. Fig. S9(a) shows the image resulting from a 685 nm centered bandpass filter with a pattern optimized for this wavelength using our method. When the emission filter is changed to be centered at 510 nm with a bandwidth of 15 nm, the resulting image acquired is shown in Fig. S9(b) using the same pattern as used for Fig. S9(a). Fig. S9(b) shows an undesirable intensity distribution among the subimages. Note that whenever the emission filter is changed, the Rectangular Aperture is adjusted to prevent the FOVs of the subimages from overlapping. For SLM operations, it is recommended to use an updated calibration curve when switching to different wavelengths, therefore as a next step, the calibration settings are updated to give a 2π phase range for 532 nm, which is close to 510 nm for the purpose of this demonstration. In this updated setting, the same pattern which is optimized for a 685 nm bandpass filter is displayed on the SLM and the resulting image acquired and shown in Fig. S9(c), still showcasing a far from ideal intensity spread. Finally, the algorithm is executed using the 532


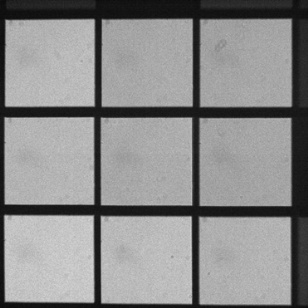

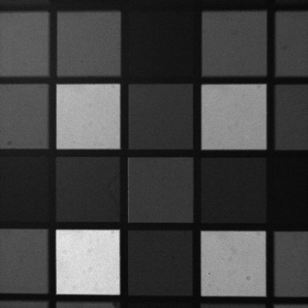


(a) (b)







(c) (d)

Fig. S9. Demonstration of wavelength dependence of the iterative optimization algorithm. (a) image resulting from optimized for 685 nm centered emission filter, acquired using 685 nm centered emission filter, with SLM calibration settings suited to 685 nm, (b) image resulting from our algorithm optimized for 685 nm centered emission filter, acquired using 510 nm centered emission filter, with SLM calibration settings suited to 685 nm, (c) image resulting from optimized for 685 nm centered emission filter, acquired using 510 nm centered emission filter, with SLM calibration settings suited to 510 nm, and (d) image resulting from in situ iteratively optimized for 510 nm centered emission filter, acquired using 510 nm centered emission filter, with SLM calibration settings suited to 510 nm.

nm based updated SLM calibration settings and deploying the 510 nm centered emission filter, and the image resulting from this optimized output pattern is shown in Fig. S9(d) which represents a much more uniform intensity distribution as compared to Fig. S9(b) and Fig. S9(c). It is recommended to deploy our optimization routine to separately acquire ideal SLM patterns for each wavelength band desired in the multifocal microscope.

8. Diffraction efficiency

The metric *M* is designed to optimize uniformity of the subimage intensities. To analyze the diffraction efficiency of the Pixelflipper, IFTA and the in situ iterative methods in our experimental system, the following procedure is executed. Note that for consistency, the optical brightness of the light source and the exposure time (set to 22 ms) in Darkfield Imaging mode is kept constant for all images presented in this section. To begin, a single reference multifocal image is acquired using a constant graylevel pattern on the SLM. For this reference image, the image intensity values are summed over a chosen square region of 80 × 80 pixels^2^ for each of the 9 subimages. In this case, the central subimage (zeroth order) receives majority of the light intensity reflecting off the SLM. The value of the intensity sum in this central subimage is denoted as $I_{s-max}$ which will be used for normalizing purposes later, while the minimum intensity sum from among the subimages in this reference image is denoted as $I_{s-min}$ which will be used for background subtraction. Next, 23 images are acquired using SLM patterns generated using each of the Pixelflipper, IFTA and our in situ iterative methods. For each image, the sum of the same 80 × 80 pixels2 for each of the 9 subimages is acquired and denoted as $\left\{ I_{s,i} \right\}$, where *i* = 1, 2, … 9. Next, we define a measure of the diffraction efficiency. Since, the diffraction efficiency is conventionally stated in terms of the throughput in a given non-zero orders, here we evaluate the throughput into all orders except for the central zeroth order. Even though we intentionally want the emission light intensity to be present in the zeroth order using our algorithm, we exempt this order, referenced as subimage 5 from the diffraction efficiency calculations since other sources (such as SLM reflective surfaces) contribute to its brightness. Therefore, the diffraction efficiency *D_e_* is defined as:

$D_{e}=\frac{sum\left\{ I_{s,i}-I_{s-min} \right\}}{I_{s-max}-I_{s-min}}\mathrm{for} i=1, 2, 3, 4, 6, 7, 8, 9,$

This *D*_e_ measure is sufficient to evaluate the relative diffraction efficiency characteristics of the three methods in this study. A plot of *D*_e_ values computed for 23 iterations of the three methods is shown in Fig. S10(a). According to Fig. S10(a), the *D_e_* values for the Pixflipper are lower compared to IFTA and in situ iterative methods. It is interesting to note that the *D_e_* for IFTA and in situ iterative methods are comparable in some cases. In particular, the data points in the marked purple square are of significance. These points indicate comparable diffraction efficiency performance for the IFTA pattern versus the in situ iterative pattern. For further analysis, the corresponding images for the IFTA data point and the in situ iterative data point inside the Fig. S10(a) marked purple square are shown in Fig. S10(b) and Fig.S10(c), respectively. Visually, the Fig. S10(b) IFTA resulting image appears relatively non-uniformly illuminated compared to the in situ iterative resulting Fig. S10(c) image. The *M* values for Fig. S10(b) and Fig. S10(c) are computed to be 0.207 and 0.448, respectively. These *M* and *D_e_* for the two cases indicate that even though both have comparable light intensities in the non-zeroth order subimages, this particluar in situ iterative method’s SLM pattern directs light away from the zeroth order to other orders travelling outside the camera’s detection region to equalize the intensity distribution among the 9 detected subimages. This uniform intensities among the subimages is more desirable than the uniformly case obtained using the IFTA pattern. The latter case could induce issues with the camera’s dynamic range, even if one scales the subimages separately. Although the current in situ iterative method implementation provides superior performance than other techniques, further study into different optimization metrics and techniques is needed to find the optimal in situ iterative metric routine to maximize both the image uniformity and diffraction efficiency.


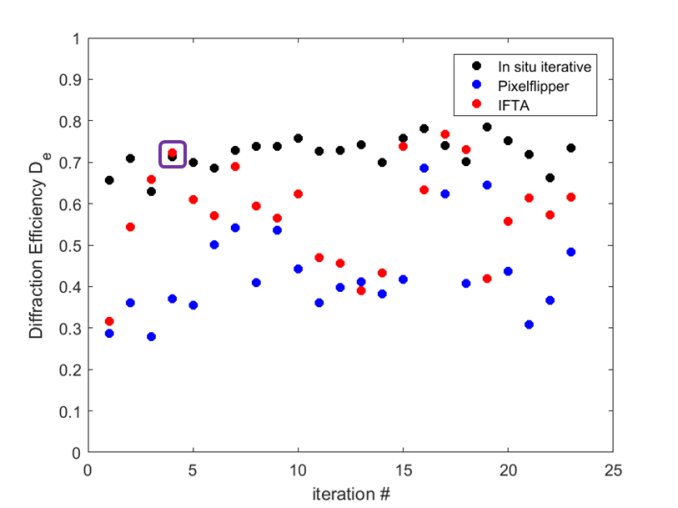


(a)


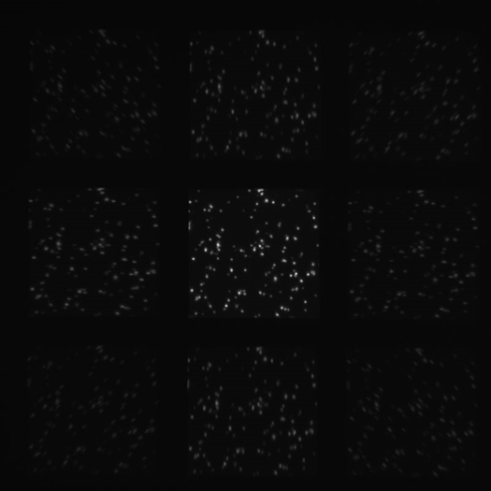

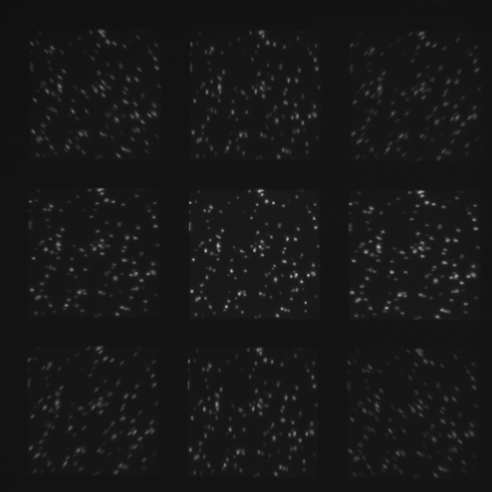


(b) (c)

Fig. S10. (a) Plot of *D_e_* values versus iteration number for images resulting from Pixelflipper, IFTA and in situ iterative methods, (b) IFTA resulting image, having *M* = 0.207, corresponding to red data point inside the marked purple square in (a), and (c) in situ iterative method resulting image, having *M* = 0.448, corresponding to red data point inside the marked purple square in (a).

9. Phase distortion function of the multifocus grating

An important part of the multifocus grating function design, after optimizing the grating unit cells to acquire even illumination of orders, is based on the detour phase effect. For a given diffraction grating of period W*g* (in units of distance), a local geometric distortion ∆x can be introduced to impart a phase shift ∆ф on the diffracted wavefront. The resulting ∆ф is a function of the diffraction order m_i_ where the subscript i represents the order number. ∆ф is expressed as:

$\Deltaф (m_{i}) = 2\pi(\frac{\Delta x}{W_{g}} )m_{i}$ Eqn. S2

Eqn. S2 can be rearranged as:

$\Delta x = (1/2\pi)W_{g}m_{i}\Deltaф$ Eqn. S3

The ∆ф expression for the microscope is derived in [3] to be:

$\Deltaф= nk\Delta z\sqrt{1-\frac{(x_{p}^{2}+y_{p}^{2})}{n^{2}f_{\mathrm{obj}}^{2}}}$ Eq. S4

where n is the refractive index of the objective immersion medium, ∆z is distance to the defocus plane from the plane of focus, $x_{p}^{2}$ and $y_{p}^{2}$ represent the pupil plane coordinates, f_obj_ is the focal length of the objective and k is the free-space wavenumber related to the emission wavelength λ via k = 2π/λ.

In the context of multifocal operation, the in-situ iterative pattern is first applied to the grating before the above mentioned phase algorithm is applied to introduce multifocal capability having ∆z > 0 nm. To calculate M values for such scenarios, correct intensity values $\left\{ I_{m,i} \right\}$ for the subimages need to be first evaluated. This is done by moving the z-stage of the sample to each plane where a given subimage is in focus where the intensity value for that particular subimage only is found. Once all 9 intensity values $\left\{ I_{m,i} \right\}$, as well as a background intensity measure $I_{b}$, are collected, M values can be computed using Eqn. (1). After computing M values using this method, we found them to be identical to the M values when there is no phase distortion (∆z = 0 nm). Therefore, we conclude that introducing phase distortion has minimal effect on the illumination uniformity of the MFM.
